# A Holistic Strategy for Classification of Sleep Stages with EEG

**DOI:** 10.3390/s22093557

**Published:** 2022-05-07

**Authors:** Sunil Kumar Prabhakar, Harikumar Rajaguru, Semin Ryu, In cheol Jeong, Dong-Ok Won

**Affiliations:** 1 Department of Artificial Intelligence Convergence, Hallym University, Chuncheon 24252, Korea; sunilprabhakar22@gmail.com (S.K.P.); sr@hallym.ac.kr (S.R.); incheol.jeong@hallym.ac.kr (I.c.J.); 2 Department of ECE, Bannari Amman Institute of Technology, Sathyamangalam 638401, India; harikumarrajaguru@gmail.com

**Keywords:** clustering, dimensionality reduction, feature extraction, selection, classification

## Abstract

Manual sleep stage scoring is usually implemented with the help of sleep specialists by means of visual inspection of the neurophysiological signals of the patient. As it is a very hectic task to perform, automated sleep stage classification systems were developed in the past, and advancements are being made consistently by researchers. The various stages of sleep are identified by these automated sleep stage classification systems, and it is quite an important step to assist doctors for the diagnosis of sleep-related disorders. In this work, a holistic strategy named as clustering and dimensionality reduction with feature extraction cum selection for classification along with deep learning (CDFCD) is proposed for the classification of sleep stages with EEG signals. Though the methodology follows a similar structural flow as proposed in the past works, many advanced and novel techniques are proposed under each category in this work flow. Initially, clustering is applied with the help of hierarchical clustering, spectral clustering, and the proposed principal component analysis (PCA)-based subspace clustering. Then the dimensionality of it is reduced with the help of the proposed singular value decomposition (SVD)-based spectral algorithm and the standard variational Bayesian matrix factorization (VBMF) technique. Then the features are extracted and selected with the two novel proposed techniques, such as the sparse group lasso technique with dual-level implementation (SGL-DLI) and the ridge regression technique with limiting weight scheme (RR-LWS). Finally, the classification happens with the less explored multiclass Gaussian process classification (MGC), the proposed random arbitrary collective classification (RACC), and the deep learning technique using long short-term memory (LSTM) along with other conventional machine learning techniques. This methodology is validated on the sleep EDF database, and the results obtained with this methodology have surpassed the results of the previous studies in terms of the obtained classification accuracy reporting a high accuracy of 93.51% even for the six-classes classification problem.

## 1. Introduction

Sleep is one of the most important functions of the brain, and it plays a vital role in a person’s life, which includes factors such as learning ability, concentration, and memory [1]. A partial or full unconsciousness is rendered by sleep to an individual, thereby making the brain a less complicated network. Conditions such as insomnia and obstructive sleep apnea are quite frequent, and they greatly affect the physical health [2]. Sleep issues cause depression, fatigue, lack of interest in academics/office, headache, frequent colds, and joint problems, and can sometimes lead even to death. A lot of road traffic accidents and fatalities are caused by drowsiness [3]. Therefore, automatic detection and analysis of sleep patterns are quite important to trace sleep-related conditions, including fatigue, drowsiness, apnea, insomnia, and so forth [3]. For the analysis of human sleep, sleep stage scoring is the gold standard, and it helps to identify the sleep stages that are important in treating sleep disorders. Based on the polysomnographic (PSG) recordings obtained from the patients during sleep time, the scoring of sleep stages is usually considered [4]. The overnight PSG recordings include electroencephalography (EEG), electrooculography (EOG), electromyography (EMG), and electrocardiography (ECG) recordings, and the visual scoring of them is performed by experts per the guidelines modelled by Rechtschaffen and Kales (R and K) [5]. The division of the PSG recording is made into a 20 or 30 s epoch, which is further classified into wakefulness (W) stage, rapid eye movement (REM) sleep, and non-REM (NREM) stages. NREM is further divided into four stages—S1, S2, S3, and S4—based on the guidelines of R and K. Multiple signal channels are included in the PSG recordings, and therefore, the visual examination of it by an expert is highly time-consuming, expensive, and prone to a lot of human errors [6]. Moreover, when the recording of the signals is performed, the sleep efficiency of the patient can be severely disturbed as the patient sleeps in an unfamiliar environment for the full night with so many adhesive electrodes and wires attached to the patient. Therefore, when analyzing these challenges, an automated sleep stage classification system is necessary as it would mitigate the time demand of the clinicians and can easily improve the diagnosis of sleep disorders. For the analysis of sleep stages, the EEG signal plays a vital role, and single/multiple EEG channels have been utilized in the past [7]. An EEG is a very efficient modality helping in acquiring the brain signals that correspond to different states from the area of scalp surface [8]. The EEG, apart from sleep analysis, helps in many other important applications, such as motor imagery classification [9], visual feedback classification [10], subject independent brain–computer interface classification [11], schizophrenia classification [12], and epilepsy classification [13]. In this work, the design of the proposed methodology is implemented only for sleep stage classification. A lot of methods and algorithms have been proposed in the past for automated sleep stage classification systems, and few important related works are discussed as follows.

A lot of analysis has been performed in many sleep-related datasets, such as sleep-EDF dataset, expanded sleep-EDF dataset, Montreal Archive of Sleep Studies (MASS) dataset, Sleep Heart Health Study (SHHS), and Massachusetts Institute of Technology–Beth Israel Hospital (MIT–BIH) dataset, ISRUC dataset, Massachusetts General Hospital (MGH), University College Dublin Sleep Apnea Database (UCD) dataset, and CAP dataset [14]. In this work, only the related works conducted on sleep-EDF are discussed as the present work was implemented only on this dataset in a very exhaustive manner. A recent survey paper published in 2020 highlighted all the previous works in the past in the field of automated sleep stage classification, which includes all the methodologies involved, algorithms implemented, classification techniques used, and so forth, thereby easing the work of other authors not to repeat the past literature over and over again [15]. However, the most recent and relevant works in the automated sleep stage classification published in recent years on sleep-EDF dataset are provided in a short manner for the readers’ elaborate understanding. It has become a fashion in recent years to use deep learning for almost all the applications in every domain, and so papers published in the past 2 to 3 years in automated sleep stage classification utilizing deep learning are discussed as follows. A convolutional neural network (CNN) design was implemented in [16,17,18,19] for automated sleep stage classification, and they reported classification accuracies of 92.5%, 84.5%, 83.6%, and 81.3%, respectively. Attention CNN produced a classification accuracy of 93.7% in [20], and a one-dimensional 1D-CNN was implemented in [21], producing a classification accuracy of 90.8% for EEG signals, 89.8% for EOG signals, and 91.2% for EEG and EOG combined signals, respectively. Elman recurrent neural network (RNN) analysis was used in [22], reporting a classification accuracy of 87.2%. A multitask CNN was utilized in [23], reporting a classification accuracy of 82.3% for EEG and EOG combined signals and 81.9% for EEG signals, respectively. A deep neural network (DNN) was implemented, reporting a classification accuracy of 86.1% in [24]. Hybrid deep learning models for automated sleep stage classification utilized CNN with bidirectional LSTM (CNN-BiLSTM) [25], CNN with bidirectional RNN (CNN-BiRNN) [26], RNN-LSTM [27], and CRNN [28] reporting classification accuracies of 82.0%, 84.3%, 86.7%, and 83.9%, respectively. Generally, the features of the signals extracted with or without dimensionality reduction and later classified by a classification procedure is the standard protocol followed. For feature extraction techniques, time domain methods, frequency domain methods, nonlinear complex methods, and so forth are utilized widely [29]. The common time domain methods used in the past for feature extraction are standard statistical methods, such as mean, variance, standard deviation, skewness, kurtosis, threshold percentile, median, Shannon entropy, Renyi entropy, zero crossing, Hjorth parameters, detrended fluctuation analysis, mutual information, and Tsallis entropy. The common frequency domain methods utilized in the past include nonparametric analysis, parametric analysis, higher-order spectra (HOS), median frequency, harmonic parameters, coherence analysis, Itakura distance, spectral entropy, and so forth [29]. The time–frequency domain methods include wavelet transform, signal decomposition, short-time Fourier transform, empirical mode decomposition, energy distribution, and Choi–Williams technique. Other nonlinear parameters involved in the past for feature extraction included correlation dimension, Lempel–Ziv complexity, Lyapunov exponent, fractal dimension, approximate entropy, sample entropy, autoregressive coefficients, phase space components, Hurst exponent, energy operators, permutation operator, and multiscale entropy [29]. Feature selection techniques involved in the past for sleep stage classification includes fuzzy C-means clustering, minimum redundancy maximum relevance, sequential techniques, artificial immune clustering, large margin neural network, fast correlation-based filter, fisher score, *t*-test, recursive feature elimination, principal component analysis, ReliefF method, and metaheuristic algorithms such as differential evolution, genetic algorithm, and particle swarm optimization (PSO) feature selection methods. Many machine learning techniques, such as linear discriminant analysis (LDA), support vector machine (SVM), artificial neural networks (ANN), naïve Bayesian classifier (NBC), quadratic discriminant analysis (QDA), K-nearest neighbor (KNN), decision trees (DT), Adaboost, K-means classifier, Gaussian mixture model (GMM), hidden Markov model (HMM), and bagging, have been utilized for sleep stage classification in the past [30]. A lot of concerns always exist with the automatic sleep stage classification system, such as deviation in the ranges of classification accuracy with sensitivity and specificity measures, careful selection of versatile feature extraction and selection methods, and execution of advanced classification techniques. Many other considerations, such as strong mathematical modelling, good computational time, high generalization and stability, and prospects for good hardware implementation in real-time situations, are also considered while developing automated sleep stage classification systems. On analyzing all the past literature, in this paper something novel was implemented, and the major contributions of the work are as follows:

(a) Initially, the clustering was implemented to EEG signals, and the clustering methodology incorporates hierarchical clustering, spectral clustering, and the proposed PCA-based subspace clustering techniques, which is the first of its kind to implement all the three techniques for EEG signal processing utilized for automated sleep stage classification.
(b) The dimensionality of the signals was then reduced with the help of the proposed SVD-based spectral algorithm and the standard VBMF. Though VBMF is already existing in the literature, very few works have been reported on its application for reducing the dimensionality of EEG, and so it is considered in this work along with the proposed SVD-based spectral technique.
(c) The features were extracted and selected with the help of techniques, such as the proposed sparse group lasso technique with dual-level implementation (SGL-DLI) and the proposed ridge regression technique with limiting weight scheme (RR-LWS). Both these two developed novel techniques have been successfully utilized in our work.
(d) Finally, classification happens with the less explored multiclass Gaussian process classification (MGC), the proposed RACC method, and the deep learning technique using LSTM, and the performance is compared with the other conventional machine learning techniques too.

**Figure 1:**
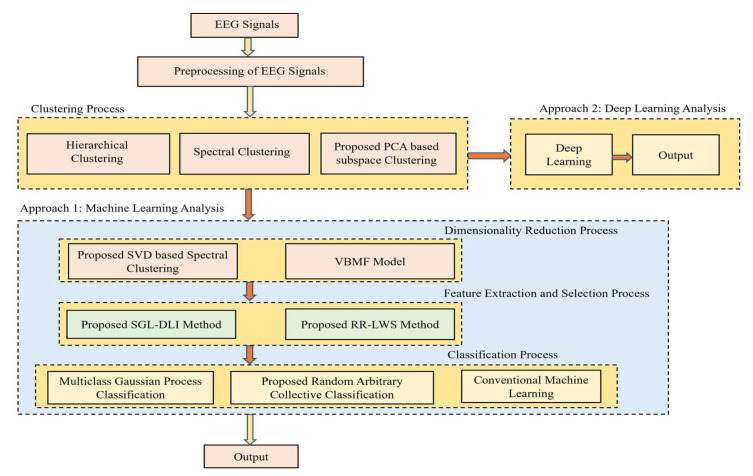
Pictorial representation of the work.

A very good mathematical modelling was provided for all the proposed techniques, and the interesting factor is the novel convergence of all the proposed techniques, which makes the whole paper in general very interesting and easy to perform the experiment and provide better results than the previous works. The workflow of the methodology is shown in Figure 1. 

The organization of the work is as follows: In Section 2, the clustering and dimensionality reduction techniques are discussed, followed by the usage of feature extraction and selection techniques in Section 3. Section 4 discusses the usage of classifiers, followed by the results and discussion in Section 5 and ended with the conclusion in Section 6.

## 2. Clustering and Dimensionality Reduction Techniques

### 2.1. Clustering Techniques

The primary task of assimilating a group of objects in such a manner in which the objects in the same cluster/group are quite similar to each other compared with those present in the other cluster/group is called clustering. In this work, hierarchical clustering, spectral clustering, and the proposed PCA-based subspace clustering are utilized for the clustering, and they are applied to the signals once the preprocessing is done with the help of independent component analysis (ICA).

#### 2.1.1. Hierarchical Clustering

One of the famous and strong manifestations of the curse of dimensionality problem is that the points considered from high dimensional distributions are quite far from their nearest neighbors, and to address the noise and outliers associated with it becomes a huge challenge [31]. In order to model the low-dimensional structure, various assumptions are imposed on the data such that the clusters should be drawn from the affine subspaces. Spectral clustering usually takes place when the cluster shape is unknown or when it deviates severely from the linear structure. With respect to the geometry of the clusters considering the noise and outliers, this clustering seems to be a very popular and effective approach. An initial distance or a similarity measure is required by the spectral clustering as the operation of it is performed on a graph constructed between the neighbors assessed and the weights dependent on such distances. In the procedure of assessing the groupings within the data and based on these groupings, the assigning of labels to the data points without supervision is performed, and the procedure is termed clustering. In some circumstances, it can perform well, but in some other circumstances, it can never perform well as we have learned with K-means clustering, fuzzy C-means clustering, and so forth [31]. Statistical assumptions are usually placed on the data so that a good performance assessment is provided. The most famous clustering technique is K-means along its variants and is utilized with many feature extraction techniques for EEG signal processing. However, in this paper, a different attempt to utilize other kinds of clustering is implemented, and the notations utilized for the clustering concept are as follows: The data points to the clusters are denoted by $X={\left\{x_{i}\right\}}_{i=1}^{n}\subset {ℜ}^{d}$, the intrinsic dimension of cluster sets is expressed by d, the number of clusters is denoted by K, and the discrete data clustering is denoted by ${\left\{X_{l}\right\}}_{l=1}^{K}$. The discrete noise data are represented as X, and the denoised data are represented as $X_{N}$. The number of points that remains in the cluster is denoted as $n_{\min}$, and W represents the weight matrix. The arbitrary value is represented by ρ. A family of clusters is built at distinct hierarchical levels by the hierarchical clustering algorithm [31]. The initiation of the individual points is performed as their own clusters, and then the merging of it is performed in an iterative manner unless it reaches a stopping criterion, and therefore, the algorithms are agglomerative. The merging of the clusters at a certain iteration is determined by the agglomerative techniques by utilizing a clustering dissimilarity metric $\rho_{c}$. For two clusters, $C_{i},C_{j}$, $\rho_{c}\left(C_{i},C_{j}\right)$ implies that the clusters are strong candidates for merging purposes. For every data point in X, let the metric be expressed as $\rho_{X}$, and along with the standard $\rho_{c}$, the corresponding clustering techniques include:

$$\rho_{SL}\left(C_{i},C_{j}\right)=\min_{x_{i}\in C_{i},x_{j}\in C_{j}\rho_{X}\left(x_{i},x_{j}\right)},\;\mathrm{single}\;\mathrm{linkage}\;\mathrm{clustering}$$

$$\rho_{CL}\left(C_{i},C_{j}\right)=\max_{x_{i}\in C_{i},x_{j}\in C_{j}\rho_{X}\left(x_{i},x_{j}\right)},\;\mathrm{complete}\;\mathrm{linkage}\;\mathrm{clustering}$$

#### 2.1.2. Spectral Clustering

To define an embedding of the data, a spectral decomposition of a Laplacian matrix is utilized, and then the embedded data are clustered using a standard algorithm called K-means. On the data, a weighted graph is constructed that can specify the local relationships. For the points that are far apart from each other, the graph has very low edge weights. For the points that are very close to each other, the graph has very high edge weights. Then the partitioning of the graph is performed into clusters so that small edge weights are present between each cluster and large edge weights are within each cluster. A kernel function is denoted here as $f_{\sigma }:ℜ\to \left[0,1\right]$ with a specific scale parameter σ. Assume that $W_{ij}=f_{\sigma }\left(\rho \left(x_{i},x_{j}\right)\right)$ is the respective weight matrix for a given metric $\rho :{ℜ}^{D}\times {ℜ}^{D}\to \left[0,\infty \right)$ and some discrete set $X={\left\{x_{i}\right\}}_{i=1}^{n}\subset {ℜ}^{D}$. The degree of point $x_{i}$ is expressed as $d_{i}=\sum_{j=1}^{n}W_{ij}$, and the diagonal degree matrix $D_{i1}=d_{i}$, $D_{ij}=0$ is also defined for i≠j. By expressing L=D−W, the graph Laplacian is defined and is normalized to get the symmetric Laplacian $L_{SYM}=I-D^{-\frac{1}{2}}WD^{-\frac{1}{2}}$. It can also be normalized to obtain a random walk Laplacian $L_{RW}=I-D^{-1}W$. To define an embedding, utilizing the eigenvectors of L leads to un-normalized spectral clustering, while the eigenvector of $L_{SYM}$ leads to normalized spectral clustering. The within-cluster similarity is always maximized by normalized spectral clustering and is generally utilized in practice.

The spectral clustering with $L_{SYM}$ is considered, and then the spectral embedding is constructed [32]. In order to indicate the matrix $L_{SYM}$ computed on the data set X, $L_{SYM}\left(X,\rho ,f_{\sigma }\right)$ is utilized effectively with a metric ρ and kernel $f_{\sigma }$. The eigenvalues of $L_{SYM}$ are specified by $\lambda_{1}\leq \ldots \leq \lambda_{n}$, and the respective eigenvectors are denoted by $\phi_{1},\ldots ,\phi_{n}$. The data have to be clustered into K groups, and initially, a n×K matrix Φ is formed where columns are expressed by ${\left\{\phi_{i}\right\}}_{i=1}^{k}$, where the K eigenvectors are called as the K principal eigenvectors. To obtain matrix V, the normalization of the rows of Φ is performed and is expressed as:

(1) Vij=Φij|(ΣjΦij2)12

The rows of V are specified by ${\left\{v_{i}\right\}}_{i=1}^{n}\in {ℜ}^{K}$. If $g:{ℜ}^{D}\to {ℜ}^{K}$ is implemented to specify the spectral embedding, then $v_{i}=g\left(x_{i}\right)$. At the end, the clustering of ${\left\{v_{i}\right\}}_{i=1}^{n}$ is performed into K groups by applying K-means where the partition of data points ${\left\{x_{i}\right\}}_{i=1}^{n}$ is expressed. Similarly, $L_{RW}$ can be utilized. A vital aspect of spectral clustering is to choose K. In order to estimate the total number of clusters as the largest empirical eigenmap, the eigenvalues of $L_{SYM}$ are often used and are represented as $\hat{K}=\arg\max_{i}\lambda_{i+1}-\lambda_{i}$. It should also be observed that $\lambda_{\hat{k}+1}-\lambda_{\hat{k}}$ is maximal and also $\lambda_{i}$ should be close to zero for $i\leq \hat{k}$. The spectral clustering algorithm utilized in this work is given in Algorithm 1. When utilizing a sparse Laplacian especially, where the sparse nearest neighbor graph is defined by W, this algorithm can be utilized.
**Algorithm 1:** Spectral clustering process with a metric ρ.Input: (data) ${\left\{x_{i}\right\}}_{i=1}^{n}$, (kernel function) $f_{\sigma }$, and (scaling parameter) σ>0
Output: Y(labels) with clustered values
 1. The weight matrix $W\in {ℜ}^{n\times n}$ is computed with $W_{ij}=f_{\sigma }\left(\rho \left(x_{i},x_{j}\right)\right)$. 2. The diagonal degree matrix $D\in {ℜ}^{n\times n}$ is computed with $D_{ii}=\sum_{j=1}^{n}W_{ij}$. 3. The symmetric normalized Laplacian $L_{SYM}=I-D^{-\frac{1}{2}}WD^{-\frac{1}{2}}$ is formed. 4. The eigendecomposition ${\left\{\left(\phi_{k},\lambda_{k}\right)\right\}}_{k=1}^{n}$ is computed and sorted so that $0=\lambda_{1}\leq \lambda_{2}\leq \ldots \leq \lambda_{n}$. 5. The number of clusters K is estimated as: $\hat{K}=\arg\max_{k}\lambda_{k+1}-\lambda_{k}$. 6. The row normalized spectral embedding is defined by $v_{i}={\left(\phi_{1}\left(x_{i}\right),\phi_{2}\left(x_{i}\right),\ldots ,\phi_{\hat{k}}\left(x_{i}\right)\right)/\left\|\phi_{1}\left(x_{i}\right),\phi_{2}\left(x_{i}\right),\ldots ,\phi_{\hat{k}}\left(x_{i}\right)\right\|}_{2}$ for 1≤i≤n. 7. By implementing K-means on the data ${\left\{v_{i}\right\}}_{i=1}^{n}$, the labels Y are computed by utilizing $\hat{K}$ as the total number of clusters, thereby implementing the concept of clustering successfully.

#### 2.1.3. Proposed PCA-Based Subspace Clustering

Subspace clustering is an important technique, and the mainstream approach has two important phases, such as calculation of the affinity matrix, followed by the application of spectral clustering [33]. To enhance the scalability of sparse subspace clustering, many techniques have been proposed in the literature. A random subset of the whole dataset in clustering is considered, and then it utilizes these clusters to group the output of sample data points. When the random subset is small or large, this technique can be scaled well. From the raw dataset X, which has N observations of various parameters as input, the clustering assignment for each point is considered an output in the dataset. In the sample clustering stage, a subset $\tilde{X}$ is drawn from n≪N points. The $\left(d_{\max}+1\right)$ nearest neighbor points in X is found out for every point ${\tilde{x}}_{i}\in \tilde{X}$. The index set of these points is denoted by $C_{i}$. Therefore, the subclustering of ${\tilde{x}}_{i}$ is called $Xc_{i}$. The affirmative matrix D is computed by each element ${\left[D\right]}_{ij}$ and is nothing but the similarity computed between $Xc_{i}$ and $Xc_{j}$. By eliminating the spurious connections with the implementation of principal component analysis (PCA), the affinity matrix is sparsified [34]. With the sparsified affinity matrix, the spectral clustering on $\tilde{X}$ is conducted. To the clustered points in $\tilde{X}$, a classifier is fit, and the points may be classified, but in our case, the dimensionality of it is still reduced, and the best features are extracted and selected so that a very good classification accuracy can be obtained at a later stage. A total of n subclusters are formulated with the help of the sampled dataset. These n subclusters are divided and grouped into k clusters. The linear model of subspaces is central to the concept of clustering. Around every sampled point, the neighborhood of points is computed by applying a thresholding based on the similarity score of inner products. There is huge dependence on the self-representative property of linear subspaces here, and therefore, the concept of distance in between the subclusters is developed to build an affinity matrix. The proposed PCA-based subspace clustering is expressed in Algorithm 2 as follows.
**Algorithm 2**: Proposed PCA-based subspace clustering.Input: Data X, number of subspaces k, sampling size n, regularization parameter $\lambda_{1}$ and $\lambda_{2}$, neighborhood threshold $d_{\max}$, residual minimization parameter m, affinity threshold $t_{\max}$.Output: The label vector l of all points in X with clustered values.
 1. The uniform sampling of n points $\tilde{X}$ from X is performed. 2. The subclusters are constructed. 3. Implement PCA on the subclusters. 4. An affinity matrix is constructed. 5. The adjacency matrix is sparsified.
  For $j=1\begin{array}{cc} to & n\begin{array}{cc} & do \end{array} \end{array}$
  $w:={\left[D\right]}_{j}$  For $i=1\begin{array}{cc} & to\begin{array}{cc} & n\begin{array}{cc} & do \end{array} \end{array} \end{array}$  If ${\left[D\right]}_{ij}\leq w\left(n-d_{\max}\right)$, then    ${\;\;\;|D|}_{ij}:=0$   End  End 6. Cluster $\tilde{X}:set\begin{array}{cc} D:=D+D^{T} & \end{array}$. 7. Sample points in $\tilde{X}$ are clustered by implementing spectral clustering on D. 8. Indicate the labels of $\tilde{X}$ by $l_{in}$. 9. The label of the entire dataset X is obtained by combining $l_{in}$ and $l_{out}$ so that the entire l can be obtained and the clustering is performed successfully.


### 2.2. Dimensionality Reduction Techniques

To reduce the overall dimension, dimensionality reduction techniques are highly useful, and techniques incorporated here are the proposed SVD-based spectral algorithm and the standard variational Bayesian matrix factorization technique. Once the clustering of the signals is done using the above three techniques, the dimensionality of it is reduced so that the aim of achieving a high classification accuracy is achieved later.

#### 2.2.1. SVD-Based Spectral Algorithm

Here in this approach, an undirected graph G=([n],E) is initially assembled along with an unknown vector $r\in {ℜ}^{n}$, where the score related with node i is expressed as $r_{i}$ for the obtained clustered values. The assumption of G is considered G(n,p), where the edges between the vertices have a huge independence with a probability p. For each i, the assumption is that $r_{i}$ is handled uniformly, $r_{i}\in \left[0,M\right]$. Therefore, $r_{i}-r_{j}\in \left[-M,M\right]$ for all i,j. M is not considered to be known to the algorithm. A noisy and independent measurement $R_{ij}$ is obtained for every {i,j}∈E as

(2) $$R_{ij}=\{\begin{array}{c} r_{i}-r_{j};w.p\eta \\ ∼U\left[-M,M\right];w.p\begin{array}{cc} & \left(1-\eta \right) \end{array} \end{array}$$

In order to control the noise level, the parameter η∈[0,1] is used, and the indication of the noise level in an explicit manner is performed by γ=1−η. The parameters η and p are not considered to be known to the algorithm.

The measurement matrix $H\in {ℜ}^{n\times n}$ is formed by initializing the following conditions:

(3) $$H_{ij}=0,\forall_{i}=1,\ldots ,n$$

(4) $$H_{ij}=R_{ij}\;\mathrm{and}\;H_{ji}=-R_{ij},\;\mathrm{if}\;(i,j)\in E$$

(5) $$H_{ij}=0,\;\mathrm{if}\;\left(i,j\right)\notin E$$

where $R_{ij}$ denotes the independent measurements.

The main intention is to recover the score vector r and also to recover the ranking π, which is induced by r. The complete graph G along with the noise-free measurement conditions makes $H=re^{T}-er^{T}$, which is nothing but a rank 2 skew-symmetric matrix. By specifying $\alpha =\frac{r^{T}e}{n}$, it can be understood that the two nonzero left singular vectors are $u_{1}=e/\sqrt{n},u_{2}=\frac{r-\alpha e}{{\left\|r-\alpha e\right\|}_{2}}$ with equal nonzero singular vector $\sigma_{1}=\sigma_{2}={\left\|r-\alpha e\right\|}_{2}\sqrt{n}$ [35].

Training a vector orthonormal to $e/\sqrt{n}$ is important for any orthonormal basis for span $\left\{u_{1},u_{2}\right\}$ so that the candidate solutions $\pm \frac{r-\alpha e}{{\left\|r-\alpha e\right\|}_{2}}$ are obtained. In order to recover the scale information of r, these candidates are multiplied by $\sigma_{1}/\sqrt{n}$. By selecting the best candidate that has high consistency among the measurements, the resolving of the sign ambiguity is performed easily. Therefore, ranking and synchronization is implemented here as it is a famous spectral technique to recover the ranks and scores of items. The application of SVD for ranking and synchronization is expressed in Algorithm 3 as follows.
**Algorithm 3**: SVD for ranking and synchronization.Input: Measurement graph G=([n],E) and pairwise measurement $R_{ij}$ for {i,j}∈E assigned for the clustered values.Output: Rank estimates: $\hat{\pi }$ and score estimates $\hat{r}\in {ℜ}^{n}$ considered as the dimensionally reduced values.
 1. Measurement matrix formation $H\in {ℜ}^{n\times n}$ by utilizing $R_{ij}$. 2. Trace the top 2 left singular vectors of H, namely, ${\hat{u}}_{1}$, ${\hat{u}}_{2}$. 3. As an orthogonal projection of $u_{1}=e/\sqrt{n}$ onto space $\left\{{\hat{u}}_{1},{\hat{u}}_{2}\right\}$,obtain vector ${\bar{u}}_{1}$. 4. Unit vector ${\tilde{u}}_{2}\in span\left\{{\hat{u}}_{1},{\hat{u}}_{2}\right\}$ is obtained. 5. Rank recovery: induced by ${\tilde{u}}_{2}$, the ranking $\tilde{\pi }$ is obtained. 6. Minimize the number of upsets and reconcile its global sign. 7. The ranking estimate $\hat{\pi }$ is found out. 8. Score recovery: To recover the scale τ∈ℜ, ${\tilde{u}}_{2},H$ is utilized and the output is expressed, giving the dimensionally reduced values as:
$$\hat{r}=r{\tilde{u}}_{2}-\frac{e^{T}\left(T{\tilde{u}}_{2}\right)}{n}e$$



#### 2.2.2. Variational Bayesian Matrix Factorization

In order to uncover a low-rank latent structure of data, matrix factorization is utilized, where a product of two factor matrices is obtained by approximating the data matrix [36]. For the purposes of collaborative prediction, the most famous technique utilized is matrix factorization, where the user and item factor matrices are used to predict the unknown ratings; therefore, the approximation of a user-item matrix as their respective product can be analyzed well. Assuming that $Z\in {ℜ}^{P\times Q}$ indicates a user-item rating matrix, $Z_{pq}$ of the (p,q) entry indicates the user rating p on item q. The factor matrices $U=\left[u_{1},\ldots ,u_{P}\right]\in {ℜ}^{K\times P}$ and $V=\left[v_{1},\ldots ,v_{Q}\right]\in {ℜ}^{K\times Q}$ are determined by the matrix factorization so that the rating matrix Z is approximated by $U^{T}V$.

(6) $$Z≃U^{T}V$$

Here, the rank of the factor matrices is denoted by K.

The regularized squared error loss is minimized and expressed as:

(7) $$\sum_{(p,q)\in \Omega }\left[{\left(Z_{pq}-u_{p}^{T}v_{q}\right)}^{2}+\lambda \left({\left\|u_{p}\right\|}^{2}+{\left\|v_{q}\right\|}^{2}\right)\right]$$

where a collection of indices of the observed entries in Z is represented by Ω and the regularization parameter is represented by λ. By alternating the stochastic gradient descent techniques or the least squares, the solving of the problem (7) can be performed in an efficient manner. The metaparameters, such as learning rate, regularization parameters, and the total number of iterations, should be carefully tuned so that the overfitting on the training data is avoided. All the model parameters are integrated so that the overfitting problem is alleviated successfully by means of the implementation of the Bayesian concept on matrix factorization. Thus, without the need for more parameter tuning, the learning of the complex models can be conducted easily. The side information can be easily incorporated by the Bayesian matrix factorization by implementing the Gaussian priors on user and item factor matrices. To the respective side information, each prior can be repressed so that the time and space complexity is reduced. With respect to the rank K, a cubic time and quadratic space complexity is obtained by VBMF as the variational distributions are considered to be matrixwise independent. An additional cubic time and quadratic space complexity is necessary if the incorporation of the side information is performed to the VBMF depending on the feature vector size obtained by the side information. Thus, with the prohibition of the usage of rich side information, high-dimensional feature vector is achieved, and the dimensionally reduced values are obtained. In order to satisfy the element-wise independence, the full factorization of the variational distribution is performed.

## 3. Feature Extraction and Selection Techniques

Once the dimensionality is reduced, the features have to be extracted and selected, and therefore, the two techniques proposed here are the sparse group lasso technique with dual-level implementation and a ridge regression technique with limiting optimal weight scheme.

### 3.1. Proposed Sparse Group Lasso Technique with Dual-Level Implementation

To identify the significant groups and features in a simultaneous manner, the most powerful regression technique utilized is sparse group lasso (SGL). The lasso and group lasso are combined by the SGL so the sparsity can be yielded at both the individual and group feature levels [37]. SGL has been implemented in machine learning, bioinformatics, signal processing, and so forth. A two-layer feature screening technique called dual layer features is proposed here. The inactive groups and features are quickly identified by this method, and ultimately, zero coefficients are guaranteed in the solution. To deal efficiently with multiple sparsity-inducing regularities, a dual-level technique is widely used. Through the framework of Fenchel duality, the dual feasible solution of SGL is developed [38]. The upper bounds should be estimated so that an efficient dual-level technique is developed.

Assume that ${\left\|\cdot \right\|}_{1},\left\|\cdot \right\|,{\left\|\cdot \right\|}_{\infty }$ is indicated as the $l_{1},l_{2}$ and $l_{\infty }$ norms, respectively. The unit $l_{1},l_{2}$ and $l_{\infty }$ norm balls in ${ℜ}^{n}$ are denoted by $B_{1}^{n},B^{n}$ and $B_{\infty }^{n}$, respectively. For set C, assume that intC is its interior value. Assume that $\Gamma_{0}\left({ℜ}^{n}\right)$ is the class of proper close convex function on ${ℜ}^{n}$. The domain of f is the set dom f:={w:f(n)<∞}. Assume ${\left[w\right]}_{i}$ as the $i^{th}$ component for $w\in {ℜ}^{n}$. G ⊂{1,2,…,n} is considered an index set, and the corresponding subvector of w is denoted by ${\left[w\right]}_{G}\in {ℜ}^{\left|G\right|}$, where the number of elements in G is denoted by |G|.

Assume that $y\in {ℜ}^{N}$ is the response vector and $X\in P^{N\times q}$ is the matrix of features. The SGL problem is expressed here with the group information available and represented as:

(8) $$\underset{\beta \in {ℜ}^{q}}{\min}\frac{1}{2}{\left\|y-\sum_{g=1}^{G}X_{g}\beta_{g}\right\|}^{2}+\lambda_{1}+\sum_{g=1}^{G}\sqrt{ng}\left\|\beta_{g}\right\|+\lambda_{2}{\left\|\beta \right\|}_{1}$$

where the number of features in the $g^{th}$ group is represented as $n_{g}$. The predictors in the group with the respective coefficient vector $\beta_{g}$ are expressed as $X_{g}\in {ℜ}^{N\times n_{g}}$.

The positive regularization parameters are represented as $\lambda_{1},\lambda_{2}$. Without loss of generality, assume $\lambda_{1}=\alpha \lambda$ and $\lambda_{2}=\lambda$ with α>0. Equation (8), therefore, can be written as follows:

(9) $$\underset{\beta \in {ℜ}^{q}}{\min}\frac{1}{2}{\left\|y-\sum_{g=1}^{G}X_{g}\beta_{g}\right\|}^{2}+\lambda \left(\alpha \sum_{g=1}^{G}\sqrt{ng}\left\|\beta_{g}\right\|+\lambda_{2}{\left\|\beta \right\|}_{1}\right)$$

The dual problem of SGL can be obtained as follows using the Lagrangian techniques as:

(10) $$\underset{\theta }{sup}\frac{1}{2}{\left\|y\right\|}^{2}-\frac{1}{2}{\left\|\frac{y}{\lambda }-\theta \right\|}^{2}$$

such that:

(11) $$X_{g}^{T}\theta \in D_{g}^{\alpha }:=\alpha \sqrt{ng}\beta +\beta \infty ,g=1,\ldots ,G$$

The intersection of closed half spaces enables the dual feasible set of lasso. Using Fenchel’s duality theorem, the dual feasible set of SGL is analyzed well. For every $X_{g}^{T}\theta \in D_{g}^{\alpha }$, Fenchel’s duality leads to an explicit decomposition $X_{g}^{T}\theta =b_{1}+b_{2}$, where one belongs to $\alpha \sqrt{ng}\beta$ and the other belongs to B∞. The procedure for developing dual-level feature extraction and selection is expressed in Algorithm 4 as follows.
**Algorithm 4:** Procedure for developing dual-level feature extraction and selection.Estimate a region θ that has dual optimum $\theta^{*}\left(\lambda ,\alpha \right)$ of Equations (10) and (11) for a given pair of parameter values (λ,α).The two optimization problems are solved as follows:$$s_{g}^{*}=\underset{\xi_{g}}{\sup}\left\{\left\|S_{1}\left(\xi_{g}\right)\right\|:\xi_{g}\in \Xi_{g}⊇X_{g}^{T}\Theta \right\},\mathrm{where}X_{g}^{T}\Theta =\{X_{g}^{T}\theta :\theta \in \Theta \},$$
$$t_{gk}^{*}=\underset{\theta }{\sup}\left\{\left|x_{gk}^{T}\theta \right|:\theta \in \Theta \right\},\mathrm{where}x_{gk}\mathrm{is}\mathrm{the}k^{th}\mathrm{column}\mathrm{of}X_{g}$$
.The dual feature screening ensures the form as:$s_{g}^{*}<\alpha \sqrt{n_{g}}\Rightarrow \beta_{g}^{*}\left(\lambda ,\alpha \right)=0$
$t_{gk}^{*}\leq 1\Rightarrow {\left[\beta_{g}^{*}\left(\lambda ,\alpha \right)\right]}_{k}=0,$ where the optimal solution of SGL in (9) is expressed as β*(λ,α), giving the best extracted and selected features.

### 3.2. Proposed Ridge Regression Technique with Limiting Optimal Weight Scheme

For the dimensionally reduced values, a subset of samples is considered initially, and then ridge regression is trained on these local data. The local dataset is arranged into a feature matrix $X_{i}$, where every row has a sample or data point along with an outcome vector $Y_{i}$, where each entry is an outcome. The local ridge regression estimates [39] are computed as follows:

(12) $${\hat{\beta }}_{i}={\left(X_{i}^{T}X_{i}+\lambda_{i}I_{p}\right)}^{-1}X_{i}^{T}Y_{i}$$

where the regularization parameter is termed as $\lambda_{i}$. By using a weighted combination, the aggregation of them is performed so that a single-shot distributed ridge estimator is constructed as:

(13) $${\hat{\beta }}_{dist}=\sum_{i=1}^{q}w_{i}{\hat{\beta }}_{i}$$

where q represents the number of sites. By a finite sample analysis of estimation error in linear models, the distributed ridge regression can be studied well. The standard linear model is considered here as Y=Xβ+ε. For ‘n’ independent samples, the n-dimensional continuous outcome vector is represented as $Y\in {ℜ}^{n}$. X is the n×p design matrix having the values of p features for each sample. The p-dimensional vector of unknown regression coefficients is expressed as $\beta ={\left(\beta_{1},\ldots ,\beta_{p}\right)}^{T}\in {ℜ}^{T}$. In order to predict the outcome variable of future samples and to firmly estimate the respective coefficients, this technique is used. Random noise can greatly affect the outcome vector $\varepsilon ={\left(\varepsilon_{1},\ldots ,\varepsilon_{n}\right)}^{T}\in {ℜ}^{n}$. The coordinates of ε are assumed to be independent random variables with zero mean and variance $\sigma^{2}$. For estimation and prediction purposes in linear models, the ridge regression estimation is the most widely used. The ridge estimator of β is recalled as follows [39]:

(14) $$\hat{\beta }\left(\lambda \right)={\left(X^{T}X+n\lambda I_{p}\right)}^{-1}X^{T}Y$$

where λ denotes a tuning parameter. Many justifications are present in their estimation. An improved estimation can be performed as the coefficient of the ordinary least squares estimators are shrunk. Supposing that the distribution of the samples is performed across q different sites or machines, the partitioning is performed and expressed as follows:

(15) $$X=\left[\begin{array}{c} X_{1}\\ :\\ :\\ X_{q} \end{array}\right],Y=\left[\begin{array}{c} Y_{1}\\ :\\ :\\ Y_{q} \end{array}\right],$$

Therefore, for the sake of approximation in a distributed setting, ridge regression estimation is widely used. When the ridge regression is performed locally on every subset of the data, a one-shot weighting technique is given more focus, and finally, the regression coefficients are aggregated by a weighted sum. The weighting technique is utilized as it serves as a useful method of initialization to iterative techniques. Moreover, a variety of new phenomena about one-shot weights can be discovered easily. Therefore, for every dataset $X_{i},Y_{i}$, the local ridge estimators are defined with a regularization parameter $\lambda_{i}$ and are expressed as follows:

(16) $${\hat{\beta }}_{i}\left(\lambda_{i}\right)={\left(X_{i}^{T}X_{i}+n_{i}\lambda_{i}I_{p}\right)}^{-1}X_{i}^{T}Y_{i}$$

By using a weighted one-shot distributed estimation summation, the local ridge estimators are combined and expressed as:

(17) $${\hat{\beta }}_{dis\tan ce}(w)=\sum_{i=1}^{q}w_{i}{\hat{\beta }}_{i}$$

The local ridge estimators are well defined, and they are not like ordinary least squares (OLS). As the ridge estimators are biased, it is not necessary to consider whether any constraints should be added on the weights or not. The proposed algorithm works well for designs X with arbitrary covariance structures Σ. Assuming the samples distributed to be n, it is considered that there is an equal distribution of the samples. A local ridge estimator ${\hat{\beta }}_{1}$ is computed along with the local estimators ${\hat{\sigma }}_{i}^{2}$ and ${\hat{\alpha }}_{i}^{2}$ of the SNR and the noise level. The qualities necessary to find the optimal weights are $m,m^{\prime }$ and λ. The procedure of Ridge regression with limiting optimal weights is expressed in Algorithm 5 as follows.
**Algorithm 5**: Ridge regression with limiting optimal weights.Input: Data matrices $\left(n_{i}\times p\right)$ and outcomes $\left(n_{i}\times 1\right)$, $\left(X_{i},Y_{i}\right)$ distributed across q sites.Output: Distributed ridge estimator ${\hat{\beta }}_{dist}$ of regression coefficients β indicating the best features extracted and selected. 1. For i←1 to q do.   Calculate the MLE ${\hat{\theta }}_{i}=\left({\hat{\sigma }}_{i}^{2},{\hat{\alpha }}_{i}^{2}\right)$ locally.   Progress ${\hat{\theta }}_{i}$ to the global data center   End 2. Get a global estimator $\hat{\theta }=\left({\hat{\sigma }}^{2},{\hat{\alpha }}^{2}\right)=q^{-1}\sum_{i=1}^{q}{\hat{\theta }}_{i}$. 3. Tuning parameters S is chosen around the initial guess $\lambda_{0}=qp/\left(n{\hat{\alpha }}^{2}\right)$. 4. For λ∈S do.   For $i\leftarrow \begin{array}{cc} 1 & to\begin{array}{cc} & q\begin{array}{cc} & do \end{array} \end{array} \end{array}$.   Compute the local ridge estimator ${\hat{\beta }}_{i}\left(\lambda \right)={\left(X_{i}^{T}X_{i}+n_{i}\lambda I_{p}\right)}^{-1}X_{i}^{T}Y$.   The weight $w_{i}$ is computed for the $i^{th}$ local estimator as $w_{i}\left(\lambda \right)=\frac{{\hat{\sigma }}^{2}{\hat{\alpha }}^{2}\left(1-\lambda m\right)}{F+qG}$.   Progress ${\hat{\beta }}_{i}\left(\lambda \right)$ and $w_{i}\left(\lambda \right)$ to the global data center.   End   Terminate the performance of the distributed ridge estimator.   End 5. Select the best tuning parameter $\lambda^{*}$. 6. Output the respective distributed ridge estimator $${\hat{\beta }}_{dist}\left(\lambda^{*}\right)=\sum_{i=1}^{q}w_{i}\left(\lambda^{*}\right){\hat{\beta }}_{i}\left(\lambda^{*}\right)$$


The tuning parameter λ is chosen by the grid search process. Therefore, the limiting optimal weights too are estimated successfully by this algorithm, and the best features are extracted and selected through this technique.

## 4. Classification Techniques

The features extracted and selected are then fed to the classification stage. The classification techniques proposed in this work are the multiclass Gaussian process classification (MGC), the proposed random arbitrary collective classification (RACC), deep learning methods, and other standard conventional techniques. A standard 10-fold cross-validation technique was utilized for all the implemented pattern recognition and machine learning techniques.

### 4.1. Multiclass Gaussian Process Classification

The multiclass classification problems can be well addressed by Gaussian processes [40], and it is as follows: a dataset comprising N instances with $X={\left(x_{1},\ldots ,x_{N}\right)}^{T}$ as the observed explaining attributes and $y={\left(y_{1},\ldots ,y_{N}\right)}^{T}$ as the target class labels, where $y_{i}\in \left\{1,\ldots ,C\right\}$ and C>2 are the number of classes. Making predictions about the label $y^{*}$ of a new instance $x^{*}$ is the primary task of interest, given the observed data X and y. Every class label has been obtained by utilizing the labelling rule for the multiclass classification with the Gaussian process as follows:

(18) $$y_{i}=\underset{c}{\arg\max}f^{c}\left(x_{i}\right)$$

where $f^{c}(.)$, for c=1,…,C are the various latent functions, and each of them communicates with various class labels. By analyzing the latent function with the highest value at the data point $y_{i}$, the class label can be obtained. Assume $f_{i}={\left(f^{\prime }\left(x_{i}\right),\ldots ,f^{C}\left(x_{i}\right)\right)}^{T}$. The likelihood of the values of every latent function at a training point under this labelling rule is expressed by:

(19) $$p\left(y_{i}|f_{i}\right)=\prod_{c\neq y_{i}}\Theta \left(f^{y_{i}}\left(x_{i}\right)-f^{c}\left(x_{i}\right)\right)$$

where the Heaviside step function is denoted as Θ(⋅). By analyzing and marginalizing noise present around the latent functions $f^{c}\left(\cdot \right)$, other likelihood functions, such as the softmax likelihood, are considered. Around each $f^{c}$, the Gaussian noise is considered. The actual class label $y_{i}$, which is related to $x_{i}$, is considered to account for the labelling errors, and it could have been replaced with a particular probability ε so that some other class labels are reached. Therefore, the likelihood becomes as follows:

(20) $$p\left(y_{i}|f_{i}\right)=\left(1-\varepsilon \right)\prod_{c\neq y_{i}}\Theta \left(f^{\left(y_{i}\right)}\left(x_{i}\right)-f^{c}\left(x_{i}\right)\right)+\frac{\varepsilon }{C-1}\left[1-\prod_{c\neq y_{i}}\Theta \left(f^{y_{i}}\left(x_{i}\right)-f^{c}\left(x_{i}\right)\right)\right]$$

For every latent function $f^{c}\left(\cdot \right)$, the assumptions of a GP prior are performed so that a multiclass classification with GPs is addressed. Therefore, it is represented as $p\left(f^{c}\right)∼GP\left(0,k_{\theta }\left(\cdot ,\cdot \right)\right)$, where $k_{\theta_{c}}\left(\cdot ,\cdot \right)$ represents a covariance function with hyperparameter $\theta_{c}$. A famous example of covariance function includes the squared exponential covariance function and is represented as follows:

(21) $$k_{\theta_{c}}\left(x,x^{\prime }\right)=\sigma^{2}\exp\left\{-\frac{1}{2}\sum_{j=1}^{d}\frac{{\left(x_{j}-x_{j}^{\prime }\right)}^{2}}{l_{j}}\right\}+I\left[x=x^{\prime }\right]\sigma_{0}^{2}$$

where the indicator function is represented as I[⋅] and $\theta_{c}=\left\{\sigma^{2},\sigma_{0},{\left\{l_{j}\right\}}_{j=1}^{d}\right\}$, which are the hyperparameters. The length scale is represented by $l_{j}$, the amplitude parameter is represented by $\sigma^{2}$, and the level of additive Gaussian noise and $f^{c}$ is represented by $\sigma_{0}^{2}$. For each latent function $f^{c}\left(\cdot \right)$, the hyperparameter will be quite different from each other. The posterior distribution of $f={\left\{f_{i}\right\}}_{i=1}^{y}$ is computed so that the predictions about the potential class label of a new data point $x^{*}$ is made. The latent function values that are pretty compatible with the observed data are summarized by this distribution. Using Baye’s rule, the computation of the posterior distribution is performed as follows:

(22) $$p\left(f|y\right)=\frac{p\left(y|f\right)p\left(f\right)}{p(y)}=\frac{\left[\prod_{i=1}^{N}p\left(y_{i}|f_{i}\right)\right]\left[\prod_{c=1}^{C}p\left(f^{c}\right)\right]}{p\left(y\right)}$$

where $f^{c}={\left(f_{c}\left(x_{1}\right),\ldots ,f_{c}\left(x_{N}\right)\right)}^{T}$ and $p\left(f^{c}\right)=N\left(f^{c}|0,K^{c}\right)$ are a multivariate Gaussian distribution with zero mean and covariance matrix $K^{c}$ with $K_{i,j}^{c}=k_{\theta_{c}}\left(x_{i},x_{j}\right)$. The normalization constant is represented as p(y)=∫p(y|f)p(f)df and is known as marginal likelihood. In order to get good values for the model hyperparameters $\theta_{c}$, it can be maximized well. Computing the marginal likelihood is slightly difficult, and so to approximate the posterior, inference methods are utilized. A famously used inference technique is variational inference technique. The main advantage of using variational inference is that it can transform the approximate inference issue into a goal optimization problem, and it can be solved easily using stochastic optimization techniques.

### 4.2. Proposed Random Arbitrary Collective Classification

For classification tasks, a very famous framework is ensemble classification, which is nothing but a combination of the consequences of a lot of weak learners to get the ultimate classification [41]. The stability and accuracy of weak classifiers are improved greatly, always leading to a higher performance than the individual weak classifier. Here, a random arbitrary collective classification (RACC) is proposed that can be hybrid with any base classifier. The base classifier used here is SVM. RACC is thus a very flexible ensemble classification framework. Assume that the observation pair (x,y) considers values from X×{0,1}, where X denotes an open subset of ${ℜ}^{q}$, q represents a positive integer, and the class label is represented by y. A total of ‘n’ observation pairs $\left\{\left(x_{i},y_{i}\right),i=1,\ldots ,n\right\}$ are assumed in the training set. To indicate the prediction result of the classifier, $C_{n}^{S-T}\left(x\right)\in \left\{0,1\right\}$ is utilized. $B_{2}$ random subspaces ${\left\{S_{jk}\right\}}_{k=1}^{B_{2}}$ are generated from the $j^{th}$$\left(j\in \left\{1,\ldots ,B_{1}\right\}\right)$ weak learner. The optimal one $S_{j*}$ is chosen based on some criterion to be mentioned. By utilizing only a portion of training samples in this subspace $S_{j*}$, the training of the weak learner is performed. To form the decision function, the aggregation of the $B_{1}$ weak classifiers $C_{n}^{S_{1*}-T},\ldots ,C_{n}^{S_{B_{1*}}-T}$ is performed as:

(23) $$C_{n}^{RACC}(x)=1\left(\frac{1}{B_{1}}\sum_{j=1}^{B_{1}}C_{n}^{S_{j*}-T}(x)>\alpha \right)$$

where α indicates a threshold, which has to be determined. A flexible framework can be admitted here, where any selected classification techniques can act as the base classifiers, such as LDA, KNN, SVM, QDA, and DT. The ranking on the significance of variables in the $B_{1}$ subspaces ${\left\{b_{j*}\right\}}_{j=1}^{B_{1}}$ is explained by this ensemble process. The minimal discriminative set for every learner can be covered easily with this procedure, and the methodology is as follows.

Assuming that n pairs of observations are present $\left\{\left(x_{i},y_{i}\right),i=1,\ldots n\right\}\sim \left(x,y\right)\in X\times \left\{0,1\right\}$, where X denotes an open subset of ${ℜ}^{q}$, q indicates a positive integer and y={0,1} is the class label. To specify the whole feature set, $S_{FULL}=\left\{1,\ldots ,q\right\}$ is utilized. For classes 0 (*y* = 0) and 1 (*y* = 1), the marginal densities of x are assumed and expressed as $f^{\left(0\right)}$ and $f^{\left(1\right)}$. The respective probability estimates they influence are indicated as $p^{\left(0\right)}$ and $p^{\left(1\right)}$. Using the following mixture model, the joint distribution of (x,y) can be expressed as follows:

(24) $$x|y=y_{0}\sim \left(1-y_{0}\right)f^{\left(0\right)}+y_{0}f^{\left(1\right)},y_{0}=0,1$$

where the Bernoulli variable is represented as y with a success probability $\pi_{1}=1-\pi_{0}\in \left(0,1\right)$.

To express the cardinality, |S| is used for any subspace S. The probability estimate observed by the marginal distribution of x is indicated as $Q^{x}$, which is expressed as $\pi_{0}Q^{\left(0\right)}+\pi_{1}Q^{\left(1\right)}$. For classes 0 and 1, the respective marginal densities are expressed as $f_{s}^{\left(0\right)}$ and $f_{s}^{\left(1\right)}$ when they are restricted to the feature subspace S. The generation of the $B_{2}$ independent arbitrary subspaces is performed as $S_{j1},\ldots ,S_{jB2}$ so that each weak learner can be trained. Then the selection of the optimal subspace $S_{j*}$ is performed based on some important criterion, and only in $S_{j*}$, the weak learners are trained, and therefore, the $B_{1}$ weak classifiers ${\left\{C_{n}^{S_{j*}-T}\right\}}_{j=1}^{B}$ are obtained. The final decision function is obtained by means of aggregation of the outputs of ${\left\{C_{n}^{S_{j*}-T}\right\}}_{j=1}^{B}$ by computing a simple average. Algorithm 6 expresses the whole procedure in detail.
**Algorithm 6**: RACC.Input: Training data ${\left\{\left(x_{i},y_{i}\right)\right\}}_{i=1}^{n}$, new data x, type of base classifiers T, subspace distribution D, integers $B_{1}$ and $B_{2}$, criterion C.Output: Predicted label $C_{n}^{RACC}(x)$, the chosen proposition of every feature η. 1. Generate random subspaces independently, $S_{jk}\sim D,1\leq j\leq B_{1},1\leq k\leq B_{2}$. 2. For j←1 to $B_{1}$ do.   Choosing of optimal subspace $S_{j*}$ is performed from ${\left\{S_{jk}\right\}}_{k=1}^{B_{2}}$ based on C and T.   End 3. Develop the collective decision function as an ensembled one, and represent it as:$$v_{n}(x)=\frac{1}{B_{1}}\sum_{j=1}^{B_{1}}C_{n}^{S_{j*}-T}\left(x\right)$$. 4. Based on Equation (2), the threshold is set. 5. The predicted label $C_{n}^{RACC}(x)=1\left(v_{n}(x)>\hat{\alpha }\right)$ is given as output, which is the chosen proposition of every feature $\eta ={\left(n_{1},\ldots ,n_{q}\right)}^{T}$.


Hierarchical uniform distribution is used to choose the subspace distribution D. From the uniform distribution over {1,…,D}, the generation of the subspace size ‘d’ is performed. The adjustment of the subspace distribution could be performed if sufficient details are present with respect to the data structure.

### 4.3. LSTM Recurrent Network

**Figure 2:**
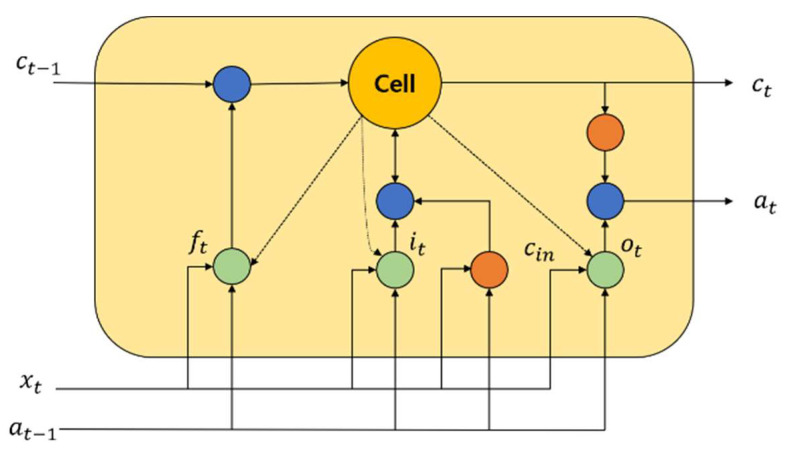
An LSTM representation.

One of the famous time recurrent neural networks is LSTM [42]. For predicting the time series of important events, LSTM is highly useful. The historical information can be easily retained by this neural network, and therefore, the learning of long-term dependence information is easily realized. An input gate, a forget gate, and an output gate are the most common gates contained in an LSTM network. In order to update and retain the historical information, a cell unit is utilized. Figure 2 shows the structure of an LSTM block.

By utilizing a simple single neuron, it helps to control the forget gate $f_{t}$ in the LSTM memory block. To enable the historical information storage, it helps to assess which information must be retained or discarded. The input gate $i_{t}$ is a part where neurons and the previous memory unit effects are used to create an LSTM block. To assess the historical information of the LSTM block, it is activated widely. Using a tanh neuron, the calculation of the candidate update content $c_{in}$ is performed. By utilizing the current candidate cell $c_{in}$, input gate information $i_{t}$, forget gate information $f_{t}$, and the previous time state $c_{t-1}$, the current time memory cell state value $c_{t}$ is computed. The generation of $o_{t}$ for the LSTM block in the current time is performed at the output gate. The amount of information about the current cell state is determined by $a_{t}$, and it is the output. The calculation of the activation of every gate along with the updation of the current cell state is performed as follows:

(25) $$i_{t}=sigmoid\left(W_{i}.\left[a_{t-1},x_{t},c_{t-1}\right]+b_{i}\right)$$

(26) $$f_{t}=sigmoid\left(W_{f}.\left[a_{t-1},x_{t},c_{t-1}\right]+b_{f}\right)$$

(27) $$o_{t}=sigmoid\left(W_{o}.\left[a_{t-1},x_{t},c_{t-1}\right]+b_{o}\right)$$

(28) $$c_{t}=f_{t}\cdot c_{t}+i_{t}\cdot c_{in}$$

(29) $$a_{t}=o_{t}.\tanh\left(c_{t}\right)$$

(30) $$c_{in}=\tanh\left(W_{c}\cdot \left[a_{t-1},x_{t},c_{t-1}\right]+b_{c}\right)$$

**Figure 3:**
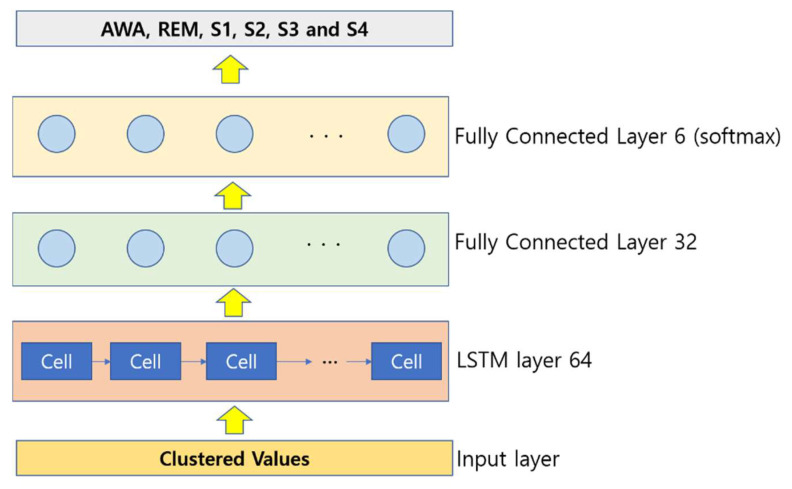
Illustration of the LSTM implementation.

For each position, the hidden vector is computed, and the last hidden vector is considered as the EEG signal representation. It is fed to a linear layer, and finally, a softmax output layer is utilized to classify the EEG. A four-layer LSTM architecture was used in this paper, which includes an input layer, an LSTM layer, and two fully connected (FC) layers. The illustration of the proposed LSTM for the EEG signal feature extraction and classification is shown in Figure 3.

#### Focal Loss

To deal with imbalanced datasets, one of the most effective ways is focal loss [43]. By transforming the cross-entropy (CE) loss function, it is obtained. The computation of CE is performed as follows:

(31) $$CE\left(\hat{y}\right)=-\log\left(\hat{y}\right)$$

A dynamically scaled CE is focal loss, where the confidence of the classification increases when the scaling factor decays to zero. The contribution of EEG examples can be automatically downweighed by this scaling factor when the model training focuses on the hard examples. The computation of FL is performed as follows:

(32) $$FL\left(\hat{y}\right)=-{\left(1-\hat{y}\right)}^{\gamma }.\log\left(\hat{y}\right),\gamma \geq 0,$$

where the modulating factor is denoted by ${\left(1-\hat{y}\right)}^{\gamma }$, and the focusing parameter is expressed by γ. When the misclassification of EEG is performed and the value of $\hat{y}$ is very small, then the value of the modulation factor is close to 1, and in such cases, the loss is barely affected. For the network parameters, optimization is important. Many kinds of gradient descent optimization algorithms are present, such as Adam, Nadam, Adagrad, and Adadelta. Here in this work, Adam is utilized.

## 5. Results and Discussion

### 5.1. Dataset Description

The sleep-EDF database contains raw physiological data having 61 data recordings considered from 42 Caucasian subjects [44,45]. The initial 39 recordings are considered from 20 healthy volunteers (SC-PSG.edf files), and they do not have any sleep-related disease. There were 10 males and 10 females, and at the time of recordings, the demographic range was between 25 to 34 years. The rest, 22 data records, were obtained from 22 participants (ST-PSG.edf files), and there were 7 males and 15 females within the demographic range of 18–79. These 22 subjects had the problem of falling asleep. Dual-channel EEG from FPz-Cz and Pz-Oz is considered in this database with a sampling rate of 100 Hz. Many other physiological signals, such as EMG, EOG, and oronasal respiration, are present in it. To understand the automatic sleep staging, dual-channel EEG data are utilized in this work as they are effective for sleep stage classification. Based on the R and K standards, the manual scoring of the 30 s epoch was performed, and the primary annotations are named as AWA, REM, S1, S2, S3, S4, ‘Movement Time’ and ‘Unscored’. Based on the R and K criteria, the total number of samples is expressed in Table 1. The total number of samples is 127,658 after movement time, and unscored categories are ignored. 

Once the clustering is done, a total of 90,000 samples are obtained, and once when dimensionality reduction is obtained, a total of 30,000 samples are obtained as the dimensionality is reduced by threefold time. When the feature extraction and selection techniques are implemented, a total of 2000 samples are selected, and finally they are fed to classification implementing a 10-fold cross-validation method. For the deep learning application model, once the clustering is performed, all the 90,000 samples are provided to it, and the classification results are obtained. The hyperparameter set for the LSTM deep learning is as follows: The number of LSTM cells is set at 64, the network layers are 4, the optimizer chosen is Adam, the dropout rate is set to 0.1 (after several trial-and-error experiments), the batch size is 128, the cost function is focal loss, and the value of focusing on parameter γ is set to 2 finally again after several trial-and-error experimentations.

Table 2 shows the results of the hierarchical clustering with SVD-based spectral algorithm dimensionality reduction technique and its performance analysis with SGL-DLI and RR-LWS for the different classifiers. When MGC is utilized, a high classification accuracy of 97.73% is obtained for two classes, 94.43% for three classes, 93.73% for four classes, 92.73% for five classes, and 92.16% for six classes under SGL-DLI technique. Similarly, when RACC is utilized, a high classification accuracy of 97.96% is obtained for two classes, 94.56% for three classes, 92.99% for four classes, 92.96% for five classes, and 92.72% for six classes under SGL-DLI technique. Similarly, when MGC is utilized, a high classification accuracy of 96.68% is obtained for two classes, 92.84% for three classes, 92.31% for four classes, 92.67% for five classes, and 91.45% for six classes under RR-LWS technique. Similarly, when RACC is utilized, a high classification accuracy of 97.55% is obtained for two classes, 91.78% for three classes, 93.56% for four classes, 91.34% for five classes, and 92.12% for six classes under RR-LWS technique. All the present results surpassed the previous results to a great extent.

Table 3 shows the results of the spectral clustering with SVD-based spectral algorithm dimensionality reduction technique and its performance analysis with SGL-DLI and RR-LWS for the different classifiers. When MGC is utilized, a high classification accuracy of 95.87% is obtained for two classes, 92.56% for three classes, 93.81% for four classes, 93.07% for five classes, and 93.51% for six classes under SGL-DLI technique. Similarly, when RACC is utilized, a high classification accuracy of 94.34% is obtained for two classes, 91.34% for three classes, 93.24% for four classes, 90.19% for five classes, and 91.20% for six classes under SGL-DLI technique. Similarly, when MGC is utilized, a high classification accuracy of 95.74% is obtained for two classes, 90.80% for three classes, 90.01% for four classes, 89.12% for five classes, and 88.75% for six classes under RR-LWS technique. Similarly, when RACC is utilized, a high classification accuracy of 95.68% is obtained for two classes, 91.01% for three classes, 90.35% for four classes, 88.38% for five classes, and 86.49% for six classes under RR-LWS technique.

Table 4 shows the results of the subspace clustering with SVD-based spectral algorithm dimensionality reduction technique and its performance analysis with SGL-DLI and RR-LWS for the different classifiers. When MGC is utilized, a high classification accuracy of 97.57% is obtained for two classes, 96.64% for three classes, 96.61% for four classes, 91.76% for five classes, and 90.49% for six classes under SGL-DLI technique. Similarly, when RACC is utilized, a high classification accuracy of 98.41% is obtained for two classes, 97.56% for three classes, 97.21% for four classes, 94.78% for five classes, and 93.12% for six classes under SGL-DLI technique. Similarly, when MGC is utilized, a high classification accuracy of 92.26% is obtained for two classes, 92.25% for three classes, 91.47% for four classes, 91.07% for five classes, and 89.23% for six classes under RR-LWS technique. Similarly, when RACC is utilized, a high classification accuracy of 96.78% is obtained for two classes, 95.97% for three classes, 94.32% for four classes, 93.89% for five classes, and 89.11% for six classes under RR-LWS technique.

Table 5 shows the results of the hierarchical clustering with VBMF dimensionality reduction technique and its performance analysis with SGL-DLI and RR-LWS for the different classifiers. When MGC is utilized, a high classification accuracy of 95.23% is obtained for two classes, 91.35% for three classes, 91.45% for four classes, 89.31% for five classes, and 90.34% for six classes under SGL-DLI technique. Similarly, when RACC is utilized, a high classification accuracy of 95.11% is obtained for two classes, 92.22% for three classes, 90.89% for four classes, 89.21% for five classes, and 92.24% for six classes under SGL-DLI technique. Similarly, when MGC is utilized, a high classification accuracy of 95.35% is obtained for two classes, 90.29% for three classes, 90.01% for four classes, 90.03 for five classes, and 90.01% for six classes under RR-LWS technique. Similarly, when RACC is utilized, a high classification accuracy of 96.11% is obtained for two classes, 90.83% for three classes, 91.09% for four classes, 89.01% for five classes, and 90.12% for six classes under RR-LWS technique.

Table 6 shows the results of the spectral clustering with VBMF dimensionality reduction technique and its performance analysis with SGL-DLI and RR-LWS for the different classifiers. When MGC is utilized, a high classification accuracy of 93.66% is obtained for two classes, 90.23% for three classes, 90.15% for four classes, 90.34% for five classes, and 89.65% for six classes under SGL-DLI technique. Similarly, when RACC is utilized, a high classification accuracy of 92.54% is obtained for two classes, 89.11% for three classes, 88.77% for four classes, 88.02% for five classes, and 87.18% for six classes under SGL-DLI technique. Similarly, when MGC is utilized, a high classification accuracy of 92.98% is obtained for two classes, 90.55% for three classes, 90.02% for four classes, 88.98% for five classes, and 87.67% for six classes under RR-LWS technique. Similarly, when RACC is utilized, a high classification accuracy of 93.09% is obtained for two classes, 89.67% for three classes, 88.11% for four classes, 86.71% for five classes, and 85.45% for six classes under RR-LWS technique.

Table 7 shows the results of the subspace clustering with VBMF dimensionality reduction technique and its performance analysis with SGL-DLI and RR-LWS for the different classifiers. When MGC is utilized, a high classification accuracy of 96.45% is obtained for two classes, 95.26% for three classes, 94.08% for four classes, 90.45% for five classes, and 90.08% for six classes under SGL-DLI technique. Similarly, when RACC is utilized, a high classification accuracy of 97.33% is obtained for two classes, 96.98% for three classes, 95.85% for four classes, 93.84% for five classes, and 92.03% for six classes under SGL-DLI technique. Similarly, when MGC is utilized, a high classification accuracy of 92.89% is obtained for two classes, 91.02% for three classes, 90.74% for four classes, 90.56% for five classes, and 88.42% for six classes under RR-LWS technique. Similarly, when RACC is utilized, a high classification accuracy of 95.01% is obtained for two classes, 94.05% for three classes, 93.13% for four classes, 92.43% for five classes, and 89.85% for six classes under RR-LWS technique.

Table 8 shows the results of the clustering methodology with deep learning LSTM method. For the two-classes classification, the classification accuracies produced are 96.35% for hierarchical clustering, 97.85% for spectral clustering, and 97.38% for subspace clustering. For the three-classes classification, the classification accuracies produced are 95.11% for hierarchical clustering, 95.99% for spectral clustering, and 96.78% for subspace clustering. For the four-classes classification, the classification accuracies produced are 94.71% for hierarchical clustering, 95.37% for spectral clustering, and 96.42% for subspace clustering. For the five-classes classification, the classification accuracies produced are 90.65% for hierarchical clustering, 93.35% for spectral clustering, and 93.22% for subspace clustering. For the six-classes classification, the classification accuracies produced are 90.42% for hierarchical clustering, 92.31% for spectral clustering, and 92.47% for subspace clustering.

### 5.2. Performance Comparison with Previous Works

The results obtained in this work are compared with the previous works and expressed in Table 9.

In the literature, there was only one recently published paper reporting results from two channels (Pz-Oz and Fpz-Cz), and so the present results were compared with it. Most of the papers have concentrated only on a single-channel EEG or EOG, and some have clubbed both EEG and EOG as reported in [21], and therefore, the current results obtained cannot be compared with them. Moreover, many results have utilized the extended version of sleep-EDF database, which has about 197 recordings released in 2018. It is a very huge database, and it is rarely analyzed as a whole dataset, and most of the reports have analyzed only a small portion or subset of it. Therefore, the current results cannot be compared with those results too as the database itself was completely different and is an extended version of the currently used database. Considering these points in mind, the hierarchical clustering with SVD-based spectral algorithm methodology with suitable classifiers produced a classification accuracy of 97.96% for two classes, 94.56% for three classes, 93.73% for four classes, 92.96% for five classes, and 92.72% for six classes. The spectral clustering with SVD-based spectral algorithm methodology with suitable classifiers produced a classification accuracy of 95.87% for two classes, 92.56% for three classes, 93.81% for four classes, 93.07% for five classes, and 93.51% for six classes, respectively. The subspace clustering with SVD-based spectral algorithm methodology with suitable classifiers produced a classification accuracy of 98.41% for two classes, 97.56% for three classes, 96.61% for four classes, 94.78% for five classes, and 93.12% for six classes. The hierarchical clustering with VBMF produced a classification accuracy of 96.11% for two classes, 92.22% for three classes, 91.45% for four classes, 90.03% for five classes, and 92.24% for six classes. The spectral clustering with VBMF produced a classification accuracy of 93.66% for two classes, 90.55% for three classes, 90.15% for four classes, 90.34% for five classes, and 89.65% for six classes. The subspace clustering with VBMF produced a classification accuracy of 97.33% for two classes, 96.98% for three classes, 95.85% for four classes, 93.84% for five classes, and 92.03% for six classes. The clustering methodology with deep learning LSTM produced a classification accuracy of 97.85% for two classes, 96.78% for three classes, 96.42% for four classes, 93.35% for five classes, and 92.47% for six classes. All the results obtained surpassed the previous results, and this shows that the present work is quite a versatile methodology. The statistical significance of the results too was analyzed. Cohen’s kappa coefficient was computed for the extracted and selected features, and the values ranged in the category of 0.6 to 1, proving that the values reached good agreement and sometimes very good agreement. The Friedman test analysis too was conducted for the process, and distinct values were obtained, proving the uniqueness in the selected features. The standard two-sided Wilcoxon test too was conducted, and the obtained ρ value was less than 0.05 in our experiment, thereby proving that a higher confidence level is achieved.

## 6. Conclusions and Future Work

Sleep disorder is a very common symptom of many neurological disorders that affects the quality of life to a great extent. Some of the common problems created due to sleep disorders are insomnia, narcolepsy, sleep-related breathing disorders, and sleep-related movement disorders. The PSG recordings of subjects are the physiological signals that are obtained during an entire night of sleep. The signal recordings, such as EEG, ECG, EOG, and EMG, are found here as PSG is a multivariate system. Once the recording is done, the scoring of sleep stages is performed on the PSG recordings by sleep experts who evaluate and grade the sleep stages. The manual determination of sleep stages is very complex and costly by means of visual inspection of PSG signals. Detecting the EEG signal variations is hard as it has a random and chaotic nature. As a result, automated sleep detection systems are developed so that the experts can be assisted well. The widely used PSG signals for the purpose of sleep stage classification are the EEG data or one or more channels. EEG is widely preferred as it is obtained using wearable technologies, and it consists of more important information. In the EEG signal processing phase, factors such as dimensionality reduction, feature extraction, and feature selection techniques are quite important, and based on that, a novel attempt to implement an interesting flow of methodology is proposed in this paper. Initially, three clustering techniques, followed by two dimensionality reduction techniques and two feature extraction cum selection techniques, were utilized and classified with around 10 classifiers to conduct an exhaustive performance analysis. Among all the results, the best results were obtained when subspace clustering with SVD-based spectral algorithm with suitable classification was performed for a two-class classification problem reporting a classification accuracy of 98.41%. Future works aim to work with many other modified versions of clustering algorithms, modified versions of dimensionality mitigation schemes, and feature extraction techniques along with plenty of other deep learning techniques to obtain a higher classification accuracy and a faster execution time with much easier applicability.

## Figures and Tables

**Table 1:** Total number of samples in sleep-EDF dataset (R and K criteria).

Number of Classes	AWA	REM	S1	S2	S3	S4
6	74,827	11,848	4848	27,292	5070	3773
5	74,827	11,848	4848	27,292	8843	
4	74,827	11,848	32,140		8843	
3	74,827	11,848	40,983			
2	74,827	52,831				

**Table 2:** Hierarchical clustering with SVD-based spectral algorithm.

	SGL-DLI					RR-LWS				
	2 Classes	3 Classes	4 Classes	5 Classes	6 Classes	2 Classes	3 Classes	4 Classes	5 Classes	6 Classes
LDA	93.24	92.34	90.62	88.24	86.34	92.67	93.67	92.45	89.67	88.35
KNN	91.32	91.26	90.67	89.32	88.36	92.32	90.83	91.78	91.13	86.16
NBC	89.92	88.11	87.13	85.92	84.65	88.98	87.56	89.43	87.84	86.56
DT	89.27	88.69	87.57	86.27	83.68	88.27	88.31	88.21	87.79	81.26
RF	87.57	85.73	84.29	82.57	81.82	86.76	84.69	85.44	84.54	84.68
Adaboost	88.32	85.25	83.41	83.32	82.78	89.15	86.45	84.67	83.63	83.16
SVM	96.57	93.22	92.62	91.57	90.91	95.93	94.32	94.86	92.85	90.83
MGC	97.73	94.43	93.73	92.73	92.16	96.68	92.84	92.31	92.67	91.45
RACC	97.96	94.56	92.99	92.96	92.72	97.55	91.78	93.56	91.34	92.12

**Table 3:** Spectral clustering with SVD-based spectral algorithm.

	SGL-DLI					RR-LWS				
	2 Classes	3 Classes	4 Classes	5 Classes	6 Classes	2 Classes	3 Classes	4 Classes	5 Classes	6 Classes
LDA	92.46	90.21	88.67	89.45	85.09	94.08	92.22	91.01	87.11	86.22
KNN	93.78	89.32	91.53	91.21	89.86	93.87	88.81	89.92	86.58	84.79
NBC	87.45	87.91	85.59	87.48	87.82	87.61	86.39	88.61	85.31	83.53
DT	87.32	89.72	89.87	88.98	8.34	88.25	87.41	87.78	88.69	80.16
RF	86.12	86.78	87.51	84.32	84.57	87.68	85.37	86.92	82.83	80.81
Adaboost	87.35	85.16	85.34	85.57	83.81	88.93	87.28	83.53	81.47	81.21
SVM	95.69	94.83	91.69	92.89	89.24	93.26	91.61	92.80	90.84	89.34
MGC	95.87	92.56	93.81	93.07	93.51	95.74	90.80	90.01	89.12	88.75
RACC	94.34	91.34	93.24	90.19	91.20	95.68	91.01	90.35	88.38	86.49

**Table 4:** Subspace clustering with SVD-based spectral algorithm.

	SGL-DLI					RR-LWS				
	2 Classes	3 Classes	4 Classes	5 Classes	6 Classes	2 Classes	3 Classes	4 Classes	5 Classes	6 Classes
LDA	93.34	91.01	90.11	89.09	88.21	95.11	94.21	92.34	91.11	89.89
KNN	95.57	92.43	91.24	90.01	87.65	96.87	95.58	92.52	91.36	88.36
NBC	92.97	91.99	91.67	88.81	86.79	93.62	92.97	91.67	90.87	89.72
DT	91.42	90.51	90.84	89.61	88.81	94.41	94.41	93.94	92.42	86.38
RF	92.56	92.12	91.57	88.54	88.27	94.64	93.46	92.28	92.19	88.67
Adaboost	94.89	93.95	92.82	89.34	88.75	93.98	93.87	92.41	91.82	89.93
SVM	96.42	95.58	95.43	92.72	90.43	94.73	92.54	91.86	91.34	88.67
MGC	97.57	96.64	96.61	91.76	90.49	92.26	92.25	91.47	91.07	89.23
RACC	98.41	97.56	97.21	94.78	93.12	96.78	95.97	94.32	93.89	89.11

**Table 5:** Hierarchical clustering with VBMF.

	SGL-DLI					RR-LWS				
	2 Classes	3 Classes	4 Classes	5 Classes	6 Classes	2 Classes	3 Classes	4 Classes	5 Classes	6 Classes
LDA	92.01	90.04	88.66	82.46	81.44	90.52	89.44	90.17	87.12	86.48
KNN	90.97	90.29	89.67	87.32	83.59	91.15	90.24	90.43	88.46	84.23
NBC	87.53	88.11	86.42	85.89	83.86	87.05	86.56	88.92	85.87	85.73
DT	87.73	86.75	86.31	83.26	83.31	86.53	85.14	87.36	85.63	80.56
RF	85.15	84.58	82.57	84.51	84.13	84.78	83.84	84.82	82.22	82.98
Adaboost	89.68	86.32	81.86	81.87	85.56	88.15	85.14	82.57	81.59	80.78
SVM	94.75	92.87	90.32	89.56	88.98	94.84	92.62	92.23	90.98	89.56
MGC	95.23	91.35	91.45	89.31	90.34	95.35	90.29	90.01	90.03	90.01
RACC	95.11	92.22	90.89	89.21	92.24	96.11	90.83	91.09	89.01	90.12

**Table 6:** Spectral clustering with VBMF.

	SGL-DLI					RR-LWS				
	2 Classes	3 Classes	4 Classes	5 Classes	6 Classes	2 Classes	3 Classes	4 Classes	5 Classes	6 Classes
LDA	90.09	88.87	86.11	87.02	83.22	92.25	91.04	91.01	85.12	81.47
KNN	91.03	87.31	88.36	89.49	87.45	91.04	89.56	89.24	85.45	82.32
NBC	86.23	85.65	84.89	83.23	85.66	85.09	85.78	8431	83.78	81.65
DT	85.56	86.98	85.03	85.98	83.78	85.30	84.92	84.89	81.74	79.87
RF	84.74	83.23	86.03	82.28	82.98	84.12	83.34	83.67	80.52	80.47
Adaboost	84.13	81.87	80.56	83.51	81.92	85.51	84.78	83.05	80.14	80.23
SVM	94.87	91.65	90.87	90.78	86.34	91.67	90.94	90.36	87.67	85.11
MGC	93.66	90.23	90.15	90.34	89.65	92.98	90.55	90.02	88.98	87.67
RACC	92.54	89.11	88.77	88.02	87.18	93.09	89.67	88.11	86.71	85.45

**Table 7:** Subspace clustering with VBMF.

	SGL-DLI					RR-LWS				
	2 Classes	3 Classes	4 Classes	5 Classes	6 Classes	2 Classes	3 Classes	4 Classes	5 Classes	6 Classes
LDA	92.46	90.23	90.14	88.11	87.57	94.46	93.54	91.98	90.67	88.11
KNN	94.86	91.67	90.78	89.24	87.14	95.98	94.81	91.76	90.33	87.23
NBC	91.45	90.09	90.05	88.67	87.52	92.72	91.79	90.24	90.21	87.56
DT	90.02	89.35	88.41	88.89	86.89	93.14	93.46	92.57	91.98	85.78
RF	91.64	91.78	9059	87.43	86.57	93.69	92.93	91.89	91.56	87.33
Adaboost	93.05	92.06	90.23	88.60	87.23	92.03	92.57	91.35	90.12	88.24
SVM	95.89	94.81	93.01	90.02	89.95	93.26	91.34	90.25	90.75	87.78
MGC	96.45	95.26	94.08	90.45	90.08	92.89	91.02	90.74	90.56	88.42
RACC	97.33	96.98	95.85	93.84	92.03	95.01	94.05	93.13	92.43	89.85

**Table 8:** Results of the clustering methodology with deep learning LSTM.

	2 Classes	3 Classes	4 Classes	5 Classes	6 Classes
Hierarchical clustering	96.35	95.11	94.71	90.65	90.42
Spectral clustering	97.85	95.99	95.37	93.35	92.31
Subspace clustering	97.38	96.78	96.42	93.22	92.47

**Table 9:** Comparison with previous works for two channels (Pz-Oz and Fpz-Cz).

Reference	Methodology	Number of Classes	Accuracy (%)
[46] (Pz-Oz and Fpz-Cz)	High-dimensional FFT features with SVM classifier	2	97.88
		3	94.41
		4	92.82
		5	91.73
		6	90.77
Proposed method (Pz-Oz and Fpz-Cz)	Hierarchical clustering with SVD-based spectral algorithm	2	97.96
		3	94.56
		4	93.73
		5	92.96
		6	92.72
Proposed method (Pz-Oz and Fpz-Cz)	Spectral clustering with SVD-based spectral algorithm	2	95.87
		3	92.56
		4	93.81
		5	93.07
		6	93.51
Proposed method (Pz-Oz and Fpz-Cz)	Subspace clustering with SVD-based spectral algorithm	2	98.41
		3	97.56
		4	96.61
		5	94.78
		6	93.12
Proposed method (Pz-Oz and Fpz-Cz)	Hierarchical clustering with VBMF	2	96.11
		3	92.22
		4	91.45
		5	90.03
		6	92.24
Proposed method (Pz-Oz and Fpz-Cz)	Spectral clustering with VBMF	2	93.66
		3	90.55
		4	90.15
		5	90.34
		6	89.65
Proposed method (Pz-Oz and Fpz-Cz)	Subspace clustering with VBMF	2	97.33
		3	96.98
		4	95.85
		5	93.84
		6	92.03
Proposed method (Pz-Oz and Fpz-Cz)	Clustering methodology with deep learning LSTM	2	97.85
		3	96.78
		4	96.42
		5	93.35
		6	92.47

## Data Availability

All the programming codes developed can be obtained upon request to the corresponding author.
